# Intake Frequency of Fish and Serum Levels of Long-chain n-3 Fatty Acids: A Cross-sectional Study within the Japan Collaborative Cohort Study

**DOI:** 10.2188/jea.15.211

**Published:** 2005-11-07

**Authors:** Kenji Wakai, Yoshinori Ito, Masayo Kojima, Shinkan Tokudome, Kotaro Ozasa, Yutaka Inaba, Kiyoko Yagyu, Akiko Tamakoshi

**Affiliations:** 1 Division of Epidemiology and Prevention, Aichi Cancer Center Research Institute.; 2 Department of Public Health, Fujita Health University School of Health Sciences.; 3 Department of Health Promotion and Preventive Medicine, Nagoya City University Graduate School of Medical Sciences.; 4 Department of Epidemiology for Community Health and Medicine, Kyoto Prefectural University of Medicine Graduate School of Medical Science.; 5 Department of Epidemiology and Environmental Health, Juntendo University School of Medicine.; 6 Department of Public Health, Aichi Medical University School of Medicine.; 7 Department of Preventive Medicine/Biostatistics and Medical Decision Making, Nagoya University Graduate School of Medicine.

**Keywords:** Fishes, Diet Records, Fatty Acids, Omega-3, Eicosapentaenoic Acid, Docosahexaenoic Acids

## Abstract

BACKGROUND: Several investigations have adopted self-reported intake frequency of fish as a surrogate for intake of long-chain n-3 fatty acids, for which protective effects against cancer have been suggested. Whether reported fish consumption reflects the fatty acid intake, however, has to be elucidated.

METHODS: We examined the association between intake frequency of fish and serum long-chain n-3 fatty acids (weight percentage of total fatty acids) among 1,257 control subjects (631 men and 626 women), aged 40-79 years, in case-control studies nested in the Japan Collaborative Cohort Study. All the subjects were not fasting when blood was drawn. Serum fatty acids were determined by gas chromatography.

RESULTS: In men, intake frequency of fresh fish and dried or salted fish was significantly but weakly correlated with serum levels of eicosapentaenoic (EPA), docosapentaenoic (n-3) (DPA), and docosahexaenoic (DHA) acids; the age-adjusted Spearman correlation coefficients ranged from 0.11 to 0.18. In women, fresh fish consumption was somewhat associated with serum EPA (Spearman correlation coefficient = 0.12) as was dried or salted fish consumption with serum DPA (0.11). A rising trend in geometric means of serum EPA, DPA, and DHA was found with an increasing intake frequency of fresh or dried/salted fish in both sexes. The geometric means adjusted for age and participating institution in the highest intake category were higher than those in the lowest by 7% to 40%.

CONCLUSIONS: A population with high self-reported frequency of fish intake, as a group, may have higher bioavailability of long-chain n-3 fatty acids than one with low frequency.

More evidence has emerged on the potentially protective effects of long-chain n-3 fatty acids abundant in fish against such cancers as those of the colon, breast, and prostate.^1^^-^^4^ Those fatty acids include eicosapentaenoic acid (EPA), docosapentaenoic acid (n-3) (DPA), and docosahexaenoic acid (DHA). Regarding their role in the prevention of cancer, however, significant inconsistencies remain among epidemiologic studies.^1^^-^^4^

One of the issues in these studies is the method of assessing the intake of long-chain n-3 fatty acids. Several investigations have adopted the self-reported intake frequency of fish, often assessed with a simple questionnaire, as a surrogate for fatty acid intake.^1^^,^^3^^-^^5^ Whether such reported fish consumption reflects the bioavailability of fatty acids, however, remains to be elucidated.

To assess the association of self-reported intake of fish with bioavailability of long-chain n-3 fatty acids, it is useful to examine the correlation between the blood levels of fatty acids and fish consumption.^6^^-^^11^ Nevertheless, only a few such studies^10^^,^^11^ have been conducted in Japan, one of the areas with the highest fish consumption in the world.^12^

We therefore conducted a cross-sectional study to examine the association between the intake frequency of fish with serum long-chain n-3 fatty acids among control subjects of case-control studies nested in the Japan Collaborative Cohort Study (JACC Study) for Evaluation of Cancer Risk sponsored by the Ministry of Education, Science, Sports and Culture of Japan (Monbusho), a nationwide prospective study.

## METHODS

### Study Population and Serum Samples

The potential subjects of this study were 1,319 control subjects, aged 40 to 79 years at baseline, in case-control studies nested in the JACC Study, who were enrolled in 20 study areas. These studies examined associations between the serum levels of fatty acids and the risks for lung, colorectal,^13^ pancreatic, and biliary tract cancers. At the baseline survey from 1988 through 1990, the subjects completed a self-administered questionnaire on lifestyle factors including dietary habits and also donated blood samples.^14^ All the subjects were not fasting when blood was drawn.

The dietary component of the questionnaire elicited five possible responses to the subject’s customary intake frequency of 33 items of Japanese foods or dishes: almost never, 1-2 times/month, 1-2 times/week, 3-4 times/week, and almost every day.^15^ In the questions, we did not specify the time frame such as “during the past one year”, “during the preceding month”, and so on.

For fish and its products, we observed fresh fish, steamed fish paste (‘kamaboko’ in Japanese), and dried or salted fish in the food list. Intake-frequency responses were not elicited in one area for fresh fish, three areas for steamed fish paste, and in two areas for dried or salted fish. Because no information on the intake frequency of fish and its products was available for 62 of the 1,319 potential subjects, we excluded them from the study, leaving 1,257 (631 men and 626 women) eligible for the present analysis. About half of them (47.2%, n = 593) were participants in the study area where blood was always drawn in a fasting condition. Sera were separated from the blood samples at laboratories in or near the surveyed municipalities as soon as possible after the blood were drawn. The serum of each participant was divided into three to five tubes (100 to 500 *μ*L per tube), which were then stored in deep freezers at -80°C until analyzed in 2001 and 2002.

Informed consent for participation was obtained individually from subjects, except in a few study areas where informed consent was provided at the group level after the aim of the study and confidentiality of the data had been explained to community leaders. The Ethics Committee of Medical Care and Research of Fujita Health University, the Ethical Board of Nagoya University School of Medicine, and the Ethics Committee of Juntendo University School of Medicine approved the protocol of the investigations, including the procedures used to obtain informed consent.

### Determination of Serum Fatty Acids

All the samples were analyzed in a single laboratory by a trained staff member. Lipids in 0.2 mL of serum were extracted with Folch’s solution under a nitrogen atmosphere.^16^ After methyl esterification by 0.4 M potassium methoxide and 14 weight percentage boron trifluoride methanol, total fatty acids were measured using a gas chromatograph (Shimadzu, GC17A, Kyoto, Japan) equipped with an Omegawax 250 capillary column (30 m × 0.25 mm i.d.; 0.25 *μ*m thickness; Supelco, Bellefonte, PA, USA). Peaks were determined using a flame-ionization detector and were quantified with an electric integrator (Shimadzu, CR-7A, Kyoto, Japan) using pure standard mixtures (Sigma, St. Louis, MO, USA). We adopted the weight percentage of each fatty acid in all detected fatty acids as a measurement value.

### Statistical Analysis

We computed the geometric means of serum levels of EPA, DPA, and DHA (weight % of total fatty acids) to summarize the data, since the measurement values showed a distribution skewed to lower values. The 95% confidence intervals of geometric means were estimated based on the standard errors of means for natural logarithms of measurement values. In the analyses of geometric means, the lowest category for the intake frequency of fresh fish (almost never) and the highest one for that of steamed fish paste (kamaboko) (almost every day) were merged into the adjacent category to attain a sufficient sample size. The difference in geometric means between sexes was tested by the *t* test for log_e_-transformed values. To examine the associations of intake frequency of fish or its products with serum long-chain n-3 fatty acids, Spearman correlation coefficients were calculated adjusting for age.

Geometric means adjusted for age (40-49, 50-59, 60-69, or 70-79 years) and participating institutions were estimated by applying generalized linear models^17^ to the logarithms of measurement values. To test for linear trends in crude or adjusted geometric means, we coded each level of age or the intake frequency of fish or its products as 0, 1, 2, 3, or 4, and then incorporated it into the generalized linear model as a single variable. All p values were two-sided, and all analyses were performed using the Statistical Analysis System^®^, release 8.2 (SAS Institute Inc., Cary, NC).^17^

## RESULTS

**Figure 1.**
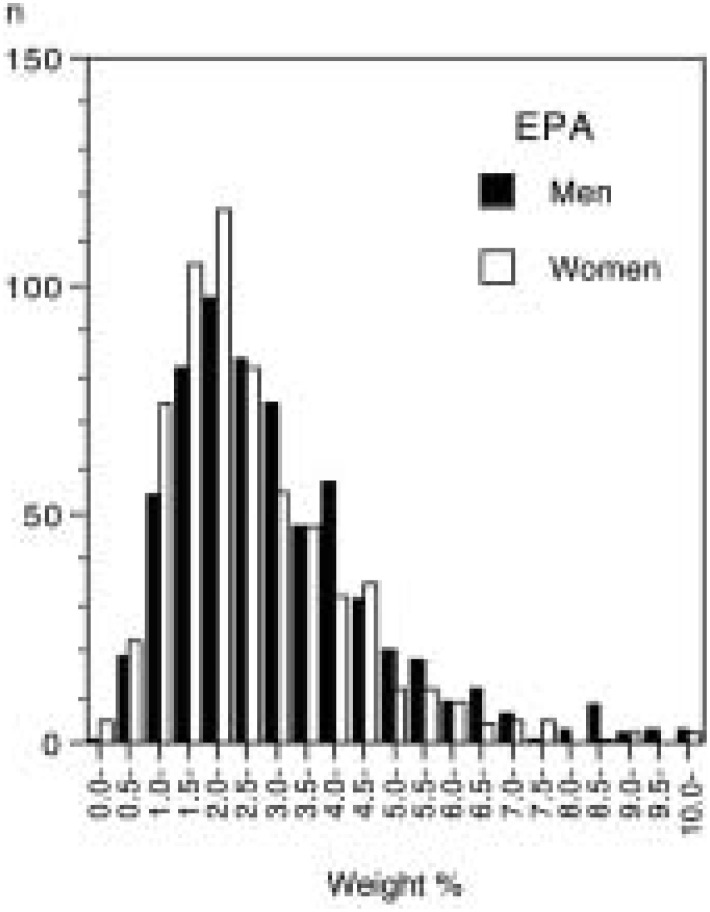
Distribution of serum level of eicosapentaenoic acid (EPA) by sex (weight % of total fatty acids).

**Figure 2.**
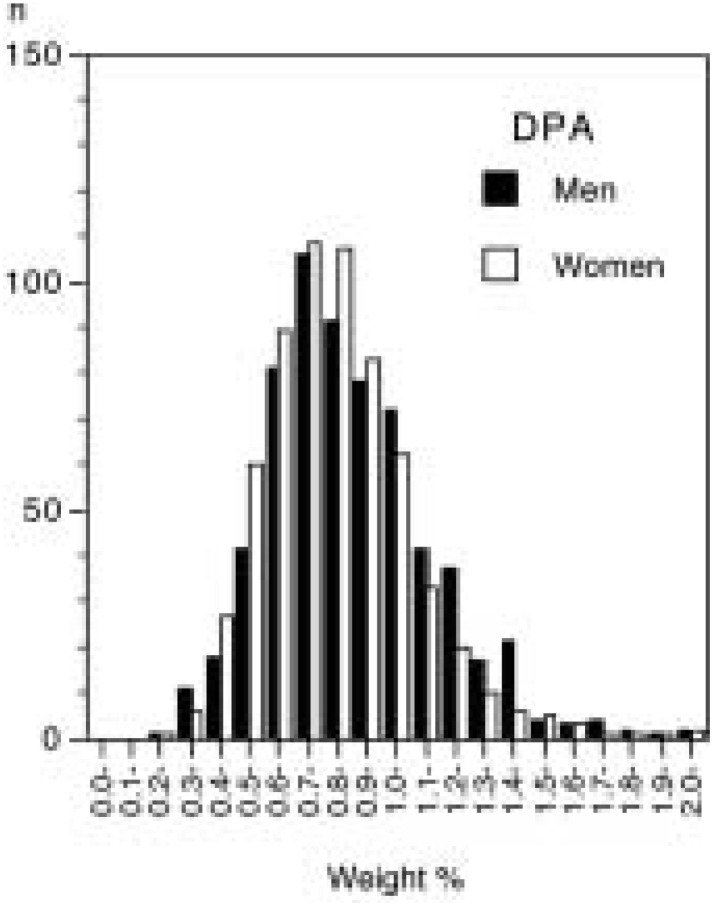
Distribution of serum level of docosapentaenoic acid (n-3) (DPA) by sex (weight % of total fatty acids).

**Figure 3.**
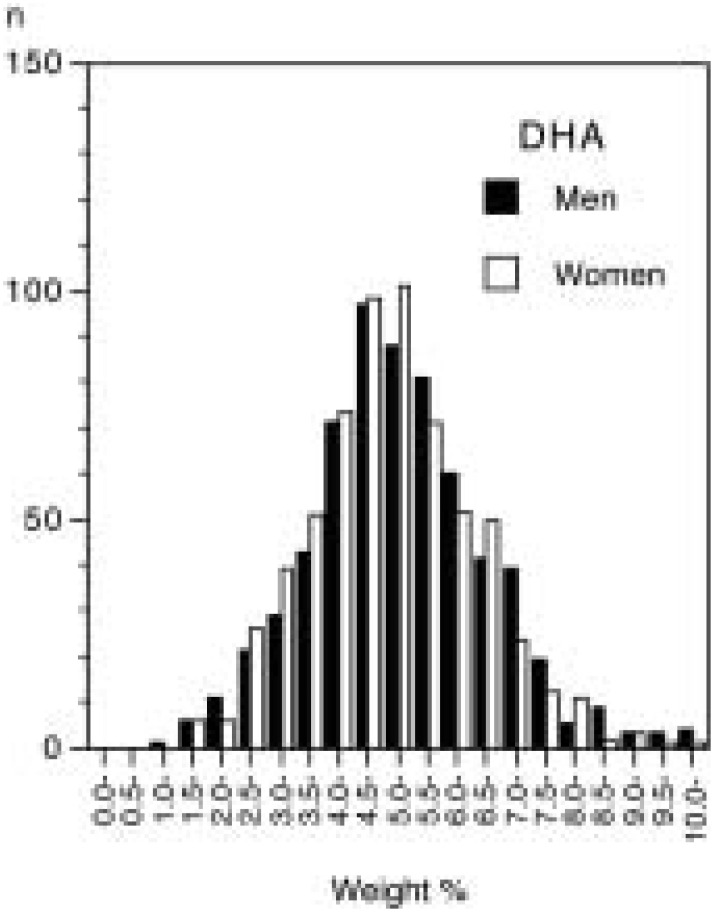
Distribution of serum level of docosahexaenoic acid (DHA) by sex (weight % of total fatty acids).

The mean age ± SD of the subjects were 63.7 ± 7.6 years in men and 63.2 ± 8.0 years in women. The distribution of serum percentages for EPA, DPA, and DHA was skewed to lower values, particularly for EPA (Figures 1 to 3). The medians were 2.84% (inter-quartile range 2.00%-4.10%) for EPA, 0.86% (0.70%-1.06%) for DPA, and 5.22% (4.38%-6.14%) for DHA in men, while they were 2.41% (1.77%-3.57%), 0.81% (0.68%-0.97%), and 5.08% (4.25%-5.98%), correspondingly, in women. The geometric means of serum levels of the long-chain n-3 fatty acids were slightly higher in men than in women (Table 1). All the means peaked at the fifties and then declined with increasing age.

**Table 1.** Geometric means (GM) of serum levels of long-chain n-3 fatty acids by sex and age (weight % of total fatty acids).

	n	EPA		DPA		DHA	
		
		GM	95% CI	GM	95% CI	GM	95% CI
Sex							
Men	631	2.82	2.71 - 2.94	0.85	0.83 - 0.88	5.07	4.95 - 5.19
Women	626	2.47	2.37 - 2.58	0.80	0.79 - 0.82	4.93	4.81 - 5.04
			p < 0.001		p < 0.001		p = 0.090

Age (years)							
40-49	69	2.25	1.98 - 2.55	0.79	0.74 - 0.85	4.88	4.55 - 5.23
50-59	309	2.82	2.66 - 3.00	0.88	0.85 - 0.91	5.19	5.02 - 5.37
60-69	570	2.74	2.62 - 2.86	0.83	0.81 - 0.85	5.08	4.95 - 5.20
70-79	309	2.41	2.27 - 2.56	0.79	0.76 - 0.82	4.70	4.54 - 4.85
			Trend p = 0.10		Trend p = 0.003		Trend p = 0.002

EPA: eicosapentaenoic acid

DPA: docosapentaenoic acid (n-3)

DHA: docosahexaenoic acid

CI: confidence interval

In men, the intake frequency of fresh fish and dried or salted fish was significantly correlated with serum concentrations of EPA, DPA, and DHA (Table 2). Those correlations, however, were rather weak, with Spearman correlation coefficients ranging from 0.11 to 0.18. In women, fresh fish consumption was somewhat associated with serum EPA (Spearman correlation coefficient = 0.12) as was dried or salted fish consumption with the serum DPA level (0.11). The intake frequency of steamed fish paste (kamaboko) showed no clear correlation with serum levels of long-chain n-3 fatty acids in either sex.

**Table 2.** Spearman correlation coefficients between intake frequency of fish and serum levels of long-chain n-3 fatty acids (weight % of total fatty acids) by sex.

	n	EPA	DPA	DHA
Men				
Fresh fish	621	0.16 ***	0.14 ***	0.16 ***
Steamed fish paste (*kamaboko*)	436	0.04	0.04	0.08
Dried or salted fish	595	0.16 ***	0.11 **	0.18 ***

Women				
Fresh fish	612	0.12 **	0.05	0.05
Steamed fish paste (*kamaboko*)	444	-0.01	0.05	0.03
Dried or salted fish	564	0.08 ^#^	0.11 **	0.06

Adjusted for age.

EPA: eicosapentaenoic acid

DPA: docosapentaenoic acid (n-3)

DHA: docosahexaenoic acid

#: p<0.10; **: p<0.01; ***: p<0.001.

An increasing trend in the geometric means of serum levels of EPA, DPA, and DHA (adjusted for age and participating institution) was found with an increasing intake frequency of fresh fish and dried or salted fish in both men and women (Table 3). The adjusted geometric means in the highest intake category were higher than those in the lowest by 7% to 40%. The consumption of steamed fish paste (kamaboko) also showed no association with serum EPA, DPA, and DHA levels in the analysis of geometric means. The findings for crude geometric means were not appreciably different from those for adjusted ones (data not shown).

**Table 3.** Geometric means (GM) of serum levels of long-chain n-3 fatty acids by sex and intake frequency of fish (weight % of total fatty acids).*

Intake frequency		Men								Women						
	
		n	EPA		DPA			DHA		n	EPA		DPA		DHA	
					
			GM	95% CI	GM		95% CI	GM	95% CI		GM	95% CI	GM	95% CI	GM	95% CI
Fresh fish																
2 times/month or less		57	2.48	2.18 - 2.83	0.83		0.77 - 0.90	4.79	4.45 - 5.15	41	2.10	1.78 - 2.48	0.80	0.74 - 0.88	4.67	4.31 - 5.05
1-2 times/week		188	2.71	2.49 - 2.94	0.88		0.84 - 0.92	5.09	4.86 - 5.33	195	2.33	2.13 - 2.55	0.80	0.77 - 0.84	4.96	4.75 - 5.18
3-4 times/week		204	2.94	2.71 - 3.19	0.92		0.88 - 0.97	5.48	5.24 - 5.73	213	2.50	2.29 - 2.74	0.81	0.77 - 0.85	4.98	4.77 - 5.20
Almost every day		172	3.20	2.92 - 3.49	0.95		0.91 - 1.00	5.61	5.34 - 5.90	163	2.82	2.55 - 3.12	0.86	0.82 - 0.91	5.21	4.96 - 5.47
			Trend p < 0.001		Trend p < 0.001			Trend p < 0.001			Trend p < 0.001		Trend p = 0.030		Trend p = 0.011	

Steamed fish paste (*kamaboko*)																
Almost never		103	2.88	2.60 - 3.19	0.90	0.85 - 0.95		5.35	5.08 - 5.63	66	2.33	2.02 - 2.68	0.79	0.74 - 0.85	4.90	4.58 - 5.24
1-2 times/month		148	2.87	2.61 - 3.16	0.87	0.82 - 0.91		5.28	5.03 - 5.54	152	2.44	2.19 - 2.73	0.81	0.77 - 0.86	5.04	4.78 - 5.31
1-2 times/week		121	2.77	2.49 - 3.08	0.87	0.82 - 0.92		5.31	5.03 - 5.60	144	2.44	2.18 - 2.72	0.83	0.78 - 0.87	5.07	4.81 - 5.35
3 times/week or more		64	3.03	2.67 - 3.44	0.91	0.85 - 0.97		5.59	5.25 - 5.95	82	2.35	2.05 - 2.69	0.82	0.77 - 0.88	4.97	4.66 - 5.31
			Trend p = 0.81		Trend p = 0.93			Trend p = 0.34			Trend p = 0.99		Trend p = 0.36		Trend p = 0.72	

Dried or salted fish																
Almost never		51	2.37	2.07 - 2.72	0.85	0.78 - 0.91		4.92	4.57 - 5.31	52	1.91	1.64 - 2.23	0.74	0.68 - 0.80	4.52	4.20 - 4.87
1-2 times/month		132	2.86	2.60 - 3.15	0.90	0.85 - 0.95		5.24	4.96 - 5.53	136	2.37	2.14 - 2.63	0.80	0.76 - 0.84	4.91	4.68 - 5.16
1-2 times/week		232	2.82	2.60 - 3.06	0.89	0.85 - 0.94		5.21	4.98 - 5.45	202	2.64	2.41 - 2.89	0.83	0.79 - 0.87	5.07	4.86 - 5.29
3-4 times/week		113	3.18	2.88 - 3.52	0.91	0.86 - 0.96		5.58	5.28 - 5.90	96	2.46	2.18 - 2.76	0.82	0.77 - 0.87	4.88	4.61 - 5.16
Almost every day		67	3.20	2.82 - 3.63	0.95	0.88 - 1.02		5.86	5.47 - 6.28	78	2.68	2.37 - 3.04	0.89	0.83 - 0.95	5.33	5.02 - 5.66
			Trend p < 0.001		Trend p = 0.048			Trend p < 0.001			Trend p = 0.003		Trend p < 0.001		Trend p = 0.003	

*: Adjusted for age and participating institution.

EPA: eicosapentaenoic acid

DPA: docosapentaenoic acid (n-3)

DHA: docosahexaenoic acid

CI: confidence interval

## DISCUSSION

In the present study, we found a clear increasing trend in the geometric means of serum concentrations of EPA, DPA, and DHA with the increasing self-reported intake frequency of fresh fish and dried or salted fish in both sexes. Although the correlations were rather weak, the two intake frequencies significantly correlated with serum concentrations of long-chain n-3 fatty acids, especially in men. The serum levels of EPA, DPA, and DHA in this study are comparable with those reported in another Japanese population.^10^

The blood levels of long-chain n-3 fatty acids have been related to the dietary intakes of fatty acids assessed using either dietary records^10^ or long and detailed food frequency questionnaires specifying a portion size.^6^^,^^8^^,^^9^^,^^11^ The correlations were considerably strong in Norway^8^^,^^9^ and Japan,^10^^,^^11^ where fish consumption is quite high; Pearson’s or Spearman’s correlation coefficients ranged from 0.3 to 0.6 for EPA, DHA, or total long-chain n-3 fatty acids. Our study demonstrated that the intake frequency of fresh fish and dried or salted fish assessed by responses to simple questions may be associated with blood levels of n-3 long-chain fatty acids at the group level, although the correlations between the intake frequency and serum levels were weaker than those obtained from dietary records or detailed food frequency questionnaires. The intake frequency of steamed fish paste (kamaboko) was not associated with serum long-chain n-3 fatty acids in our study, perhaps because the paste contains much less of these fatty acids than fresh, dried or salted fish.^18^ In addition, the study participants consumed steamed fish paste much less frequently than they did fresh or dried/salted fish (Table 3), which may also have resulted in the weak association of the paste with the serum fatty acids.

Tokudome et al.^19^ reported that chicken eggs provide one third of the DHA in middle-aged Japanese men and women, although fish and their products were the most important sources of EPA and DHA, the two major long-chain n-3 fatty acids. The multiple regression analysis by the same researchers, however, showed that the chicken egg was not a major determinant of the inter-individual variation in DHA intake and that more than 90% of the variation (R^2^) was explained by fish consumption.^19^ This may mean that fish consumption is the key to rank Japanese individuals by DHA intake whereas egg consumption is not. Fish intake also contributed to more than 90% of the inter-individual variation in EPA intake.^19^

To use weight percentage of each fatty acid in serum total fatty acids as a measurement value may have attenuated the correlation between intake frequency of fish and serum n-3 fatty acids, if fish consumption is correlated to serum levels of both total fat and n-3 fatty acid. The intake frequency of fresh fish, steamed fish paste, and dried or salted fish, however, was scarcely correlated with serum total fatty acids irrespective of sex; the age-adjusted Spearman correlation coefficients ranged from -0.08 to 0.03. This indicates that such confounding would be minimal.

When we repeated the analysis using the absolute value of each serum fatty acid, the correlations for fresh fish in both sexes and those for dried or salted fish in men were weakened; the Spearman correlation coefficients for fresh fish (adjusted for age) were 0.13, 0.07, and 0.09 for EPA, DPA, and DHA in men, and 0.09, 0.01, and 0.01 in women, respectively. The coefficients for dried or salted fish in men were 0.12, 0.04, and 0.10 for EPA, DPA, and DHA, respectively. The variations in the conditions and methods for blood sampling between institutions seem to have attenuated the correlations. Although the proportion of each fatty acid among total fatty acids in blood has often been linked to the risk of diseases,^20^^-^^22^ studies relating fish consumption to the absolute value of each fatty acid in blood samples collected in a uniform condition may provide meaningful information.

The strength of our study derives from the use of a biomarker and a large sample size from a population with a high level of fish consumption. Using serum samples allowed for objective measurements of fatty acid intake, taking account of inter-individual variations in bioavailability. Some methodological issues, however, need further elucidation.

First, we could not fully assess fish consumption due to limitations in the food frequency questionnaire, which specified neither the kind of fish nor the serving size. Long-chain n-3 fatty acids are abundant in fatty fish, the consumption of which is correlated with blood levels of EPA and DHA.^8^ Intakes of long-chain n-3 fatty acids may be more accurately estimated by specifying blue-skinned fish that are rich in the fatty acids, even when simple questions on intake frequency are used.

Second, to evaluate the levels of fatty acids, we used serum samples that were stored at -80°C for 11-14 years. Iso et al. examined 31 serum samples in 1990 and again in 1998.^22^ They reported no increase or decrease in the compositions of long-chain n-3 fatty acids during those eight years of storage at -80°C. Zeleniuch-Jacquotte and co-workers reported that storage for up to 12 years at -80°C effectively protected polyunsaturated fatty acids from oxidation.^23^ However, the effects of long-term storage for up to 14 years have not yet been confirmed.

Third, the conditions and procedures for blood sampling varied among participating institutions, e.g., subjects were not always fasting at the time of sampling. We therefore adopted the weight percentage of each fatty acid in serum total fatty acids as a measurement value to best take such a difference into consideration. In fact, no study area showed a distribution of serum levels of long-chain n-3 fatty acids (weight % of total fatty acids) greatly different from any other (data not shown). To assess the effect of the fasting condition, we reanalyzed the data while limiting the subjects to those in the study areas where all participants were fasting at blood draw. In men, the correlation coefficients of intake frequency of steamed fish paste with long-chain n-3 fatty acids increased (age-adjusted Spearman correlation coefficients 0.10, 0.12, and 0.19 for EPA, DPA, and DHA, respectively) compared with those in all subjects. In women, the coefficients of fresh fish increased (0.15, 0.10, and 0.10, correspondingly) while those of steamed fish paste (-0.10, -0.04, and -0.06) and dried or salted fish decreased (0.01, 0.03, and -0.09). It is difficult, however, to tell whether the change in correlations is due to the fasting condition of participants or due to the difference in study areas.

Finally, although we used serum total fatty acids as a biomarker, they reflect only a short-term (hours to weeks) intake.^24^ The intra-individual variations in fish consumption, therefore, may have attenuated the association between the customary consumption data and serum concentrations of long-chain n-3 fatty acids. Erythrocyte membrane and adipose tissue can be used as alternative samples to assess medium- and long-term intakes, respectively.^24^ The reference period of the food frequency questionnaire may also affect the correlation between self-reported intake frequency of fish and levels of long-chain n-3 fatty acids in biospecimens.

In conclusion, our study suggests that a population with high self-reported frequency of fish intake, as a group, has higher bioavailability of long-chain n-3 fatty acids than one with low frequency intake, despite the misclassifications at the individual level.
